# Synthetic Peptides Derived From *Lycosa Erythrognatha* Venom: Interaction With Phospholipid Membranes and Activity Against Resistant Bacteria

**DOI:** 10.3389/fmolb.2021.680940

**Published:** 2021-06-08

**Authors:** Pablo V. M. Reis, Vinícius M. Lima, Kelton R. Souza, Gabriele A. Cardoso, Marcella N. Melo-Braga, Daniel M. Santos, Rodrigo M. Verly, Adriano M. C. Pimenta, Vera Lúcia dos Santos, Maria Elena de Lima

**Affiliations:** ^1^ Departamento de Bioquímica, Instituto de Química, Universidade de São Paulo (USP), São Paulo, Brazil; ^2^ Departamento de Bioquímica e Imunologia, Instituto de Ciências Biológicas, Universidade Federal de Minas Gerais (UFMG), Belo Horizonte, Brazil; ^3^ Departamento de Química, FACET, Universidade Federal dos Vales do Jequitinhonha e Mucuri - Campus JK, Diamantina, Brazil; ^4^ Departamento de Bioquímica e Biologia Molecular, Campos Centro Oeste. Universidade Federal de São João Del-Rei, Diamantina, Brazil; ^5^ Departamento de Microbiologia, Instituto de Ciências Biológicas, Universidade Federal de Minas Gerais (UFMG), Belo Horizonte, Brazil; ^6^ Faculdade Santa Casa de Belo Horizonte, Programa de Pós-Graduação em Medicina – Biomedicina, Belo Horizonte, Brazil

**Keywords:** *Lycosa erythrognatha*, antimicrobial peptide, biofilm, antibiotic-resistant, LyeTxI, LyeTxI-b, LyeTxI/LyeTxI-b interaction with lipidic membranes

## Abstract

Superbugs are a public health problem, increasing the need of new drugs and strategies to combat them. Our group has previously identified LyeTxI, an antimicrobial peptide isolated from *Lycosa erythrognatha* spider venom. From LyeTxI, we synthesized and characterized a derived peptide named LyeTxI-b, which has shown significant *in vitro* and *in vivo* activity. In this work, we elucidate the interaction of LyeTxI-b with artificial membranes as well as its effects on resistant strains of bacteria in planktonic conditions or biofilms. Isothermal titration calorimetry revealed that LyeTxI-b interacts more rapidly and with higher intensity with artificial vesicles, showing higher affinity to anionic vesicles, when compared to synthetic LyeTxI. In calcein experiments, LyeTxI-b caused greater levels of vesicle cleavage. Both peptides showed antibacterial activity at concentrations of μmol L^−1^ against 12 different clinically isolated strains, in planktonic conditions, in a concentration-dependent manner. Furthermore, both peptides elicited a dose-dependent production of reactive oxygen species in methicillin-resistant *Staphylococcus aureus*. In *S. aureus* biofilm assay, LyeTxI-b was more potent than LyeTxI. However, none of these peptides reduced *Escherichia coli* biofilms. Our results show LyeTxI-b as a promising drug against clinically resistant strains, being a template for developing new antibiotics.

## Introduction

Antibiotics are among the therapeutic drugs that have had tremendous success and impact on humanity (for review Aminov, 2010). However, at the end of the 20th century, reports about microbial resistance began to emerge in hospitals and health centers. In an era of growing bacterial resistance to conventional antibiotics, the World Health Organization (WHO) reported, in the years 2014 (WHO, 2014) and 2017 (WHO, 2017), documents that recommended urgent measures to reduce and face the progress of antimicrobial resistance. In 2017, WHO released a list of the most important antibiotic-resistant pathogens (WHO, 2017), which included 12 families of antibiotic-resistant bacteria, divided into three ranges of priority for the development of new drugs. These ranges are: 1) Critical priority: *Acinetobacter baumannii* and *Pseudomonas aeruginosa* bacteria (resistant to carbapenem), Enterobacteriaceae family (resistant to carbapenem, multidrug-resistant to β-lactams); 2) High priority: *Enterococcus faecium* (resistant to vancomycin), *S. aureus* (resistant to penicillin and intermediates of vancomycin), *Helicobacter pylori* (resistant to clarithromycin), *Campylobacter* spp. (resistant to fluoroquinolones), *Salmonellae* family (resistant to fluoroquinolones), *Neisseria gonorrhoeae* (resistant to cephalosporin and fluoroquinolones); and 3) Medium priority: *Streptococcus pneumoniae* (not susceptible to penicillin), *Haemophilus influenzae* (resistant to ampicillin) and *Shigella* spp. (resistant to fluoroquinolones).

In this context, antimicrobial peptides (AMPs) have gained prominence as possible new tools against microbial resistance. AMPs show a broad spectrum of activity, high selectivity, low toxicity and low propensity to induce resistance [reviewed by Liu et al. (2021)]. These peptides have been described to act *via* two main mechanisms: membrane disruption and interaction with intracellular targets (Liu et al., 2021). AMPs are also remarkable for their high capacity to act synergistically, both with other peptides and with conventional antibiotics (Lazzaro et al., 2020). Although some resistance against AMPs has been identified, it is worth of note that it was significantly lower when compared to conventional antibiotics. However, to validate the use of AMPs as therapeutic molecules, some limitations must be overcome, such as expensive production, low stability *in vivo* and non-specific toxicity. The simplification of peptide sequences and the replacement of some amino acid residues (i.e., L for D) are some suggested measures that can reduce costs and improve the stability of these molecules. Lazzaro and collaborators (2020) highlighted the necessity of combining “the insights from the evolutionary diversification of the AMPs, their activity in the context of synergistic cocktails, and our growing understanding of how to limit resistance evolution,” which in their opinion could avoid “repeating the mistakes that have resulted in the current crisis of antibiotic resistance.”

A most recent approach to develop new active AMPs, i.e., those forming pores, as well as to understand their implications in biological activity, has involved the rational design of new peptides. Based in detail molecular dynamics (MD) of peptides, simulations can predict structure-based rational fine-tuning of their biological and functional properties. As an example, Chen et al. (2019) starting from a poly-leucine of 14 aminoacid residues designed of a minimalistic potent new AMP of four aminoacid residues (LDKA) with antibacterial activity against both Gram-positive (*S. aureus*) and Gram-negative (*E. coli* and *P. aeruginosa*) bacteria with MICs in the μM range, similar to naturally occurring AMPs. Then, taken together, the bioinformatic methods, the accessibility to data base banks and also, the increasing knowledge of natural peptides and their properties, may concur to a plethora of possibilities to obtain new active and selective drugs, among them the antibiotics against resistant microorganisms.

Aiming at investigating new promising AMPs that could act on superbugs, our group has isolated, characterized and synthesized several AMPs from *L. erythrognatha* spider venom. Among them, LyeTxI demonstrated an outstanding antimicrobial potential both *in vitro* and *in vivo* (Santos et al., 2010; Consuegra et al., 2013; Cruz Olivo et al., 2017; Melo-Braga et al., 2020). Based on LyeTxI, we developed a derivative form, named LyeTxI-b (Reis et al., 2018), which demonstrated even greater antimicrobial potential against some bacterial species, also showing an anti-cancer activity against glioblastoma cells (Abdel-Salam et al., 2019). LyeTxI-b was safe and effective for topical ocular use against resistant bacterial keratitis caused by penicillin-resistant *S. aureus* in rabbits, and was able to reduce the inflammatory process triggered by the disease (Silva et al., 2019), with no signs of ocular toxicity. This result motivated us to try to better understand the interaction and activity of these peptides in resistant *S. aureus.*

The mechanism of action of LyeTxI-b and LyeTxI has not yet been fully characterized. In this work, we evaluate the interaction of these peptides, which primary sequences and some biophysical features are described in Table 1, with biomimetic phospholipid membranes by using fluorescence spectroscopy, Isothermal Titration Calorimetry (ITC) and Dynamic Light Scattering (DLS), aiming at better understanding their interaction with bacterial membranes. It is well known that bacterial membranes have a wide diversity of amphipathic lipids that vary from species to species (Epand and Epand, 2011; Sohlenkamp and Geiger, 2015). Among these lipids, PG (phosphatidylglycerol) is one of the most common and represents 58% of the membrane lipids from *S. aureus* (Epand and Epand, 2011) and it was used in the experimental vesicles in our assays (see below).

**TABLE 1:** Structural and biophysical data of LyeTxI and LyeTxI-b.

Peptide	LyeTxI	LyeTxI-b
Sequence	IWLTALKFLGKNLGKHLAKQQLAKL^a^	IWLTALKFLGKNLGKLAKQQLAKL^b^
Molecular weight	2832.52 g/mol^a^	2737.42 g/mol^b^
N-Terminus	---	Acetyl^b^
C-Terminus	Amide^a^	Amide^b^
Secondary structure	α-helix^a^	α-helix^b^
Net charge at physiological pH 7.0	5	6.1
Ratio of hydrophilic residues/total residues	32%	33%

Data of LyeTxI and LyeTxI-b were obtained from (Santos et al., 2010)^a^ and (Reis et al., 2018)^b^, respectively. Net charge and the ratio of hydrophilic residues were calculated with the bioinformatic tool Peptide Calculator from Bachem Holding (https://www.bachem.com/knowledge-center/peptide-calculator/Accessed in March 2021).

Moreover, we expanded the knowledge on the antimicrobial activities of LyeTxI and LyeTxI-b using clinically isolated antimicrobial-resistant bacterial strains, under planktonic and biofilm conditions. We also investigated their activity on a methicillin-resistant *S. aureus* strain, evaluating the time of death, the oxidative stress and their behavior in experimental induction of antimicrobial resistance.

## Materials and Methods

### Peptide Synthesis and Estimation of Molar Concentration

The peptides were synthesized by GenOne Biotechnologies (Rio de Janeiro, RJ, Brazil): LyeTxI is deposited in RCSB PDB (7MMM) and BMRD code 30900; LyeTxI-b is deposited in RCSB PDB (6CL3) and BMRD code 30424.

Molar concentration was estimated based on the Lambert-Beer law, described in Schmid (2001), applying the formula: A = ε. c (mol L^-1^) l (cm), with *ε* being the molar extinction coefficient of the peptide, A the absorbance value at 280 nm read in a Varian Cary® 50 UV-Vis Spectrophotometer (Agilent, Palo Alto, United States), c the molar concentration (mol L-1), and l the optical path of 1 cm. The value of *ε* was calculated using the formule:ε280 (Lmol^−1^ cm^−1^) = 5,500 x nTrp.

In which, nTrp is the number of residues of the amino acid tryptophan. After determining the molar concentration, aliquots of the peptide were lyophilized and stored at −20°C in LoBind® protein tubes.

### Vesicles Preparation

Lipid vesicles were prepared as previously described (Verly et al., 2017). 1-palmitoyl-2-oleoyl-phosphatidylcholine (POPC) and 1-palmitoyl-2-oleoyl-phosphatidylglycerol (POPG) were purchased from Avanti Polar Lipids (Birmingham, AL). Appropriate amounts of POPC:POPG (3:1, mol:mol) were dissolved and mixed in dichloromethane. The solvent was removed with a rotary evaporator and the resulting thin film dried in vacuum for at least an hour to remove residual solvent. Thereafter, the phospholipid film was hydrated with 1 ml of 10 mM Tris-HCl buffer solution (pH 7.0). For calcein release experiments, the phospholipid film was hydrated with 1 ml of 10 mM Tris-HCl buffer solution (pH 7.0) containing 75 mM of calcein. The large multilamellar vesicles (LMVs) obtained were subjected to a sequence of cycles of freezing in liquid nitrogen and heating in a water bath. The dispersion of multilamellar vesicles was extruded eleven times through two 100 nm polycarbonate membranes in a LiposoFast® extrusion system (Avestin Inc., Ottawa, ON, Canada), at room temperature to form large unilamellar vesicles (LUVs). For the calcein release experiments, free calcein was removed by gel filtration through a Sephadex G50 small column equilibrated with a 10 mM Tris-HCl buffer solution (pH 7.0), containing 150 mM NaCl. Chromatography was performed at room temperature, just before the leakage experiments. The total phospholipid content of the eluted LUVs was determined by a colorimetric method as described in the literature (Stewart, 1980).

### Calcein Release Experiments

The ability of the peptides to induce membrane permeation was determined at 25°C by measuring calcein release from LUVs in a Cary Eclipse Fluorescence Spectrophotometer (Agilent, Palo Alto, United States) with excitation wavelength at 490 nm and emission wavelength at 515 nm. To carry out such analysis, 250 μL of POPC:POPG (3:1) LUVs (0.25 mM) were added to the fluorescence cuvette containing Tris-HCl buffer solution (pH 7) and the increase of calcein fluorescence as a function of time was continuously measured after the addition of different amounts of peptides (0.5, 1, 2, 4, 8, and 12 μg for LyeTxI and LyeTxI-b peptides) to the samples with final volume of 2.5 ml. The final concentrations of the peptides were 0.175; 0.35; 0.70; 1.40; 2.80 and 5.60 μmol ml^−1^ for LyeTxI and 0.18; 0.36; 0.72; 1.44; 2.88 and 5.76 μmol ml^−1^ for LyeTxI-b.

After 15 min, 10 μL of 10% Triton X-100 (v/v) solution was added to each experiment to obtain complete vesicle leakage and the maximum calcein fluorescence. The percentage of calcein release was calculated according to Eq. 1,

$$\%Leakage=\frac{I_{0}-I_{t}}{I_{0}-I_{T}}\times 100$$ (1)

where *I*_0_ is the fluorescence before addition of peptide, *I*_t_ is the measured time-dependent fluorescence after peptide addition and *I*_T_ the fluorescence after Triton X-100 addition.

### Isothermal Titration Calorimetry

ITC analyses were performed in a Malvern® VP-ITC microcalorimeter (Worcestershire, United Kingdom), at 25°C at the Laboratory of Synthesis and Structure of Biomolecules (LASEB - UFVJM). The calibration of the equipment was performed with deionized water Milli-Q® type 1. The solutions were previously degassed in a Microcal Thermovac® (Worcestershire, Kingdom) accessory from Malvern®. Each experiment consisted of 25 successive injections of 5 μL of 20 mM POPC:POPG (3:1) LUVs into the calorimetric cell filled with 1.400 ml of peptide solution (25 μM), at 300 s intervals. To eliminate the diffusion effects of the material from the syringe to the calorimetric cell, the first injection of 1 μL was discarded. Dilution experiments of LUVs were performed by injections of the samples in 10 mM Tris-HCl buffer solution (pH 8.0). The acquisition and isothermal treatments for each analysis were performed using the Microcal Origin 7.0 software for ITC. Non-linear fitting was performed by using the model of one-site binding based on the Wiseman Isotherm (Wiseman et al., 1989). Eq. 2 to obtain the partial molar enthalpy of complexation ($\Delta_{comp}H$= *dQ*/*d*[*X*]) at constant pressure.

$$\bar{\Delta_{comp}H}={\left(\frac{dQ}{d{\left[X\right]}_{tot}}\right)}_{P}=\Delta_{\mathrm{int}}H^{o}Vo\left[\frac{1}{2}+\frac{1-X_{R}-r}{2\sqrt{{\left(1+X_{R}-r\right)}^{2}-4X_{R}}}\right]$$ (2)

where *X* is the molar ratio of titrant and *M* (*X*_*R*_ = [*X*]_t_/[*M*]_t_) molar ratio of titrated at any point during the titration. The parameter *r* is the composition variable *(r = 1/[M]*_*t*_ *.K*_*app*_) and the parameters Δ_*int*_ *H* ^*o*^, *V*_*0*_ and *K*_*eq*_ are the standard complexation enthalpy, effective volume of the solution in the titration cell and apparent equilibrium constant, respectively.

### Dynamic Light Scattering/Zeta Potential

The effect of the peptides on the stability of POPC:POPG (3:1) LUVs was evaluated by measuring the hydrodynamic diameter (D_*h*_) and zeta potential (ζ), using an Dynamic Light Scattering analyzer of Malvern® Zetasizer nano ZS particles model BI-900 (Worcestershire, United Kingdom) from the Multi-user Laboratory of Research in Pharmacy (Multifar), Department of Pharmacy at UFVJM. A Malvern® model DTS1060 cuvette was used for all measurements. The D_*h*_ measurements were performed using monochromatic light (Ne 4 mW laser, *λ* 633 nm) and *ζ* at potentials greater than 500 mV. The experiments consisted in titrations of 5 μL of peptide (2.5 mM) in 500 μM LUV solution both suspended in 10 mM tris-HCl (pH 7.0) buffer solution at 25°C. Each point of the titration was determined by the average of three successive D_*h*_ measurements and five ζ measurements. All experiments were carried out in triplicates and only polydispersity index (PDI) values lower than 0.3 were considered valid.

### Antimicrobial Tests

Hospital bacterial isolates obtained from the culture collection of the Applied Microbiology Laboratory of the University CEUMA (Maranhão, São Luís, MA, Brazil) were used in the tests: two samples of carbapenem-resistant *A. baumannii*, five samples of *E. coli*, one sample of *P. aeruginosa*, two samples of *Serratia* sp. and two samples of *S. aureus* resistant to oxacillin. The samples were kept at−80°C in brain heart infusion broth (BHI) (Kasvi, Italy) with 10% glycerol (v/v). The samples were grown under aerobic conditions, on BHI Agar (Kasvi, Italy) for 24 h at 37°C. The procedures for assessing the susceptibility profile of microorganisms were performed according to CLSI (Clinical and Laboratory Standards Institute), based on the document M07A10 (CLSI, 2015), with modifications. To assess the minimum inhibitory concentration (MIC), LyeTxI and LyeTxI-b were diluted to decreasing concentrations from 44.8 to 0.35 µmol L^−1^ and 46.8–0.368 µmol L^−1^, respectively, in Mueller Hinton broth (MH). As a negative control, only MH broth was used, and as a positive control, the respective broths plus the inoculants. Commercial antibiotics Chloramphenicol, Gentamicin, Cephalexin and Ciprofloxacin were used as controls. The final cell concentration was 10^5^ UFC.ml^−1^ (bacteria) and 10^3^ UFC.ml^−1^ (yeast), with the plates incubated at 37°C for 24 h. The minimum inhibitory concentration (MIC) was defined as the lowest concentration at which there is apparent growth.

### Kinetics of Growth and Time-Dependent Killing

*S. aureus* [20] was grown in MH broth (10^5^ cells ml^−1^) with LyeTxI and LyeTxI-b at concentrations of one time the MIC value (LyeTxI = 1.4 µmol L^−1^; LyeTxI-b = 0.72 µmol L^−1^) and four times the MIC value, at 37°C. At time intervals (0, ½, 1, 2, 4, 6 h), aliquots of 10 µL were removed for cell viability determination by the microdrop technique (Naghili et al., 2013). Then, the suspensions were subjected to serial dilutions (10^−1^, 10^−2^ and 10^−3^) and aliquots of 5 µL were plated on BHI agar. The plates were incubated for up to 12 h, and the number of colonies per drop was counted and multiplied by the reciprocal of the dilution. The result was expressed as colony forming units (CFU). Experiments were performed in triplicate.

### Quantitation of Reactive Oxygen Species

To evaluate if the mechanism of action of LyeTxI and LyeTxI-b involves the induction of oxidative stress, we used the methodology of Jakubowski and Walkowiak (2015), with modifications. The fluorescence probe 2′, 7′-Dichlorofluorescein diacetate (Invitrogen, Life Technologies, Carlsbad, CA, United States) was used to measure intracellular ROS. Bacteria were grown in MH broth overnight with agitation, harvested and washed with phosphate buffered saline (PBS). Cells were resuspended until a final density of 10^5^ cells ml^−1^ in PBS. The experiment was carried out in a 96-well plate in triplicate with the final volume of 200 µL, of which 20 µL were of *S. aureus* [20] cells, 160 µL of LyeTxI (179.2–0.35 µmol L^−1^) and LyeTxI-b (184.32–0.36 µmol L^−1^) and 20 µL of 2′,7′-Dichlorofluorescein (10 µmol L^−1^). The plates were incubated at 37°C for 1 and 3 h. Fluorescence was measured on the Varioskan™ LUX multimode microplate reader (Thermo Fisher Scientific Inc., Waltham, MA, United States) using excitation and emission wavelengths of 488 and 520 nm, respectively. The negative control consisted in wells containing only bacterial inoculum, 2′,7′-dichlorofluorescein diacetate and PBS. The positive control included bacterial inoculum, dichlorofluorescein diacetate and 10 mM hydrogen peroxide. The ROS release percentage values were calculated taking into account the positive control as 100% release.

### Biofilm Eradication Assay

One strain of *S. aureus* [20] was submitted to the determination of minimum biofilm eradication concentrations (MBEC) with the peptides LyeTxI and LyeTxI-b as previously described (Silva et al., 2019). The bacterial suspensions (10^5^ UFC ml^−1^) were added to 96-well plates with MH medium supplemented with 1% glucose and incubated in a horizontal shaking plate at 37°C, for 24 h, for biofilm formation. The growth medium was discarded and biofilms were washed with sterile saline. After that, LyeTxI (179.2–0.35 µmol L^−1^) and LyeTxI-b (184.32–0.36 µmol L^−1^) at different concentrations, as indicated, were added. The incubation was at 37°C for 24 h. *S. aureus* [20] biofilms were resuspended, and resazurin solution (0.1 g/L) was added. The plates were incubated at 37°C for 20 min in the absence of light. Then, the plates were read in Varioskan TM (λex 570 nm and λem 590 nm) and MBEC was determined as the lowest concentration of peptide that prevented biofilm formation. Resazurin is a non-fluorescent blue and non-toxic dye, indicator of oxidation-reduction, and is used for the evaluation of cell growth (Sarker et al., 2007). When reduced to resorufin by oxidoreductases within viable cells, resazurin becomes pink and fluorescent. The level of reduction can be quantified by spectrophotometer. Positive and negative controls were submitted to the same procedures. In the positive control, there was no addition of any peptide or inhibitory drug, only the vehicle (Saline solution 0.9% NaCl). The negative control did not have any bacteria and the value was normalized to zero in all analyses. MBEC assays were performed in triplicate. Statistical analyses were performed using the Graphpad Prism program and the data expressed as mean ± standard deviation, followed by simple variance analysis (one-way ANOVA) with Turkey post-test. Results were considered significant for values of *p* < 0.05.

### Experimental Induction of Antimicrobial Resistance

Bacterial resistance was evaluated using two approaches. In the first, the bacteria were cultivated in a medium supplemented with sub-inhibitory concentrations (1/2 MIC) of LyeTxI and LyeTxI-b and transferred, at 24 h intervals, to a new medium, for 20 times. For the tests, *S. aureus* cells at exponential phase were diluted to 10^5^ CFU ml^−1^ in MH broth and incubated at 37°C under constant agitation (Xu et al., 2006; Ling et al., 2015). At the end of each passage, cells were cultured on mannitol agar medium (Kasvi, Italy) without the presence of the peptides for 16 h to quantify a possible survival of resistant bacteria. The MIC value was determined by broth microdilution, as previously described in the Antimicrobial Tests section. In the second approach, an increase in the concentration of the peptides was made at each sequential passage. The first passage was performed with sub-inhibitory concentrations (1/16 MIC) of LyeTxI and LyeTxI-b in 10^5^ CFU ml^−1^ of bacteria. At 24 h intervals, the successive passages were performed with a 100 x dilution of the previous culture, with the concentration of peptides increasing two times in each passage (Fehri et al., 2005; Xu et al., 2006). After this selection process, the bacteria were plated on mannitol agar medium without the peptides, for 16 h, and the same procedures mentioned above were performed.

## Results and Discussion

### Calcein Release From Phospholipid Vesicles

Our group has previously demonstrated that both LyeTxI and LyeTxI-b present a conformational behavior commonly observed for linear cationic antimicrobial peptides, which usually present significant conformational flexibility in aqueous medium, but adopt more ordered secondary structures in contact with membranes (Santos et al., 2010; Reis et al., 2018). In addition, previous studies revealed that LyeTxI was able to disturb the membrane integrity of POPG-containing vesicles, causing a release of encapsulated calcein (Santos et al., 2010). In the present work we used POPC:POPG (3:1 mol:mol) vesicles encapsulating calcein to check if LyeTxI-b would present any difference in the lytic effect compared with LyeTxI.

**FIGURE 1:**
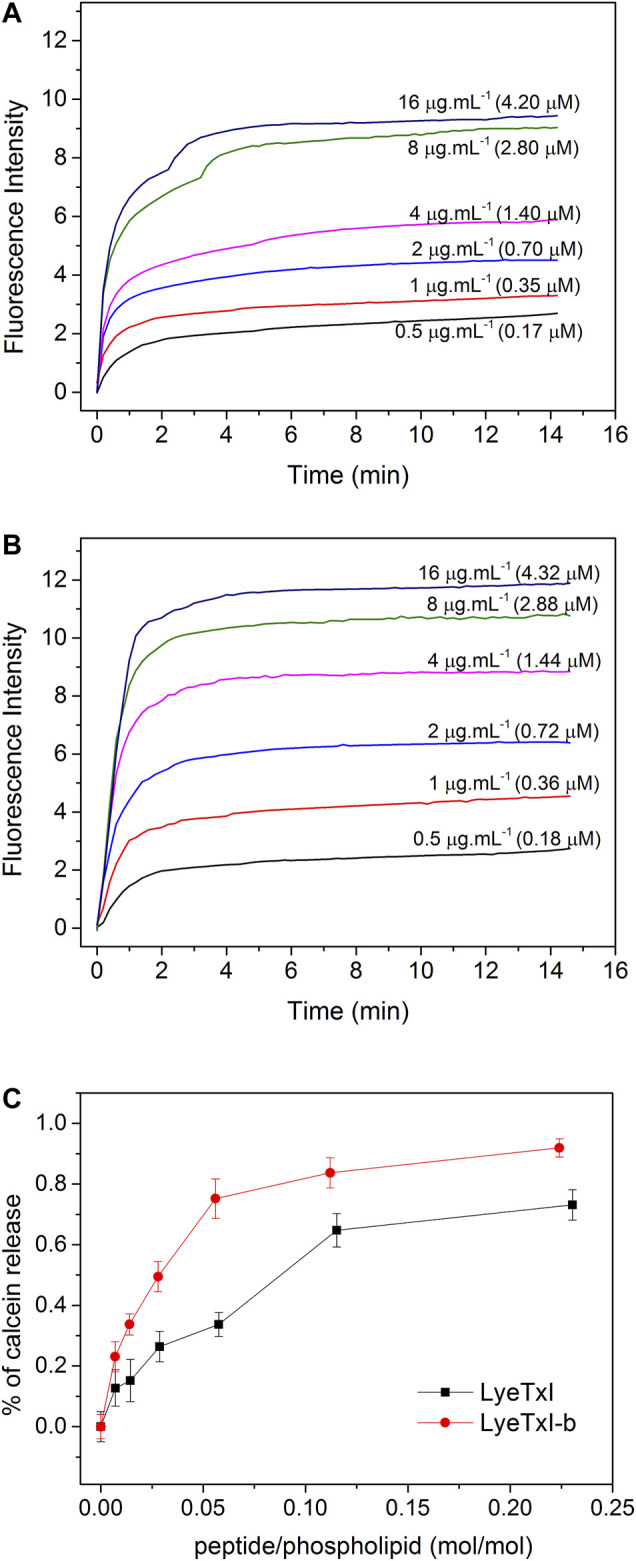
Release of calcein encapsulated in 25 mM POPC:POPG LUVs (3:1, mol:mol), at 25°C, induced by different concentrations of **(A)** LyeTxI and **(B)** LyeTxI-b. **(C)** Percentage of calcein released from LUVs in the presence of LyeTxI and LyeTxI-b.

As shown in Figure 1, a higher fluorescence intensity was observed for all LyeTxI-b concentrations (Figure 1B) when compared to the equivalent amount of LyeTxI (Figure 1A). The lytic activity of both peptides can be better compared by the ratio of lysis *versus* the peptide/phospholipid molar ratio (Figure 1C). When incubated with vesicles encapsulating calcein, LyeTxI-b was able to promote a maximum calcein percentage release of about 90%, while the maximum calcein percentage released by LyeTxI was near 70%. The lytic activity of both peptides was apparently dose-dependent, which is possibly related to a cooperative process, showing the need of an accumulation of peptides on the membrane surface for the lysis process to occur (Takeuchi et al., 2004).

### Isothermal Titration Calorimetry

**FIGURE 2:**
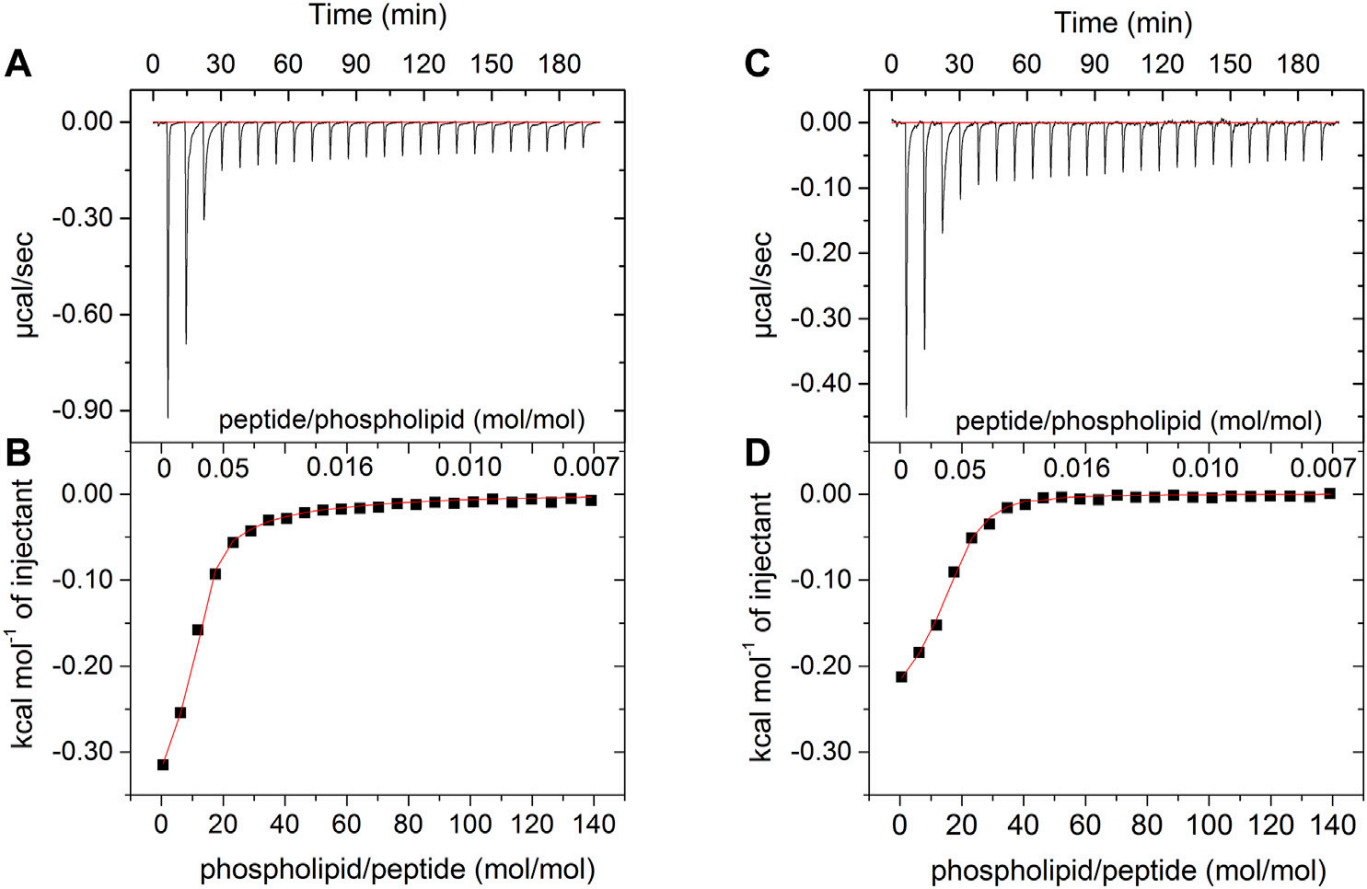
Calorimetric titration isotherms obtained from the titration of 25 μM solution of **(A,B)** LyeTxI-b and **(C,D)** LyeTxI, with 20 mM LUVs POPC:POPG (3:1, mol:mol). **(A)** and **(C)** panels show heat flow graphs for each LUVs injection as a function of time. **(B)** and **(D)** panels show the enthalpy as a function of phospholipid/peptide molar ratio for each titration and the red curves consist in the regression line generated by fitting the isotherm to a single-site peptide-membrane interaction model, according to Eq. 2 (see item *Isothermal Titration Calorimetry* section).

Figure 2 shows the results of ITC for both peptides in the presence of POPC:POPG (3:1) LUVs. Each sign of the calorimetric curve corresponds to a change in the heat flow after the injection of 5 μL of vesicles to the calorimetric cell containing the peptide solutions. For both LyeTxI and LyeTxI-b, the observed negative heat flow indicates a predominance of an exothermic process for the peptide-membrane interaction. The increase in the phospholipid/peptide molar ratio resulted in a reduction in the heat flow (Figures 2A,C), which became similar to the dilution heat of the LUVs, suggesting a high partition coefficient of the peptides with anionic vesicles (Lee et al., 2018). The respective isotherms (Figures 2B,D) correspond to the integration of the heat flow raw data for each injection as a function of the phospholipid/peptide molar ratio. The first injections of LUVs in the peptide solution revealed a stronger peptide-membrane interaction for LyeTxI-b when compared to LyeTxI, indicating a higher affinity of LyeTxI-b with the LUVs. The thermodynamic parameters of the peptide-membrane interactions were calculated by non-linear fitting (red curve in the panels B and D) and the results are presented in the Table 2. Exergonic processes were observed for the interaction of both peptides with the anionic LUVs (Seelig, 2004; Veiga et al., 2004), but a higher interaction constant (*k*_app_) of LyeTxI-b with POPC:POPG vesicles (7.270 × 10^3^ L mol^−1^) was observed when compared to LyeTxI (3.090 L mol^−1^). Consequently, LyeTxI-b interacts with more phospholipid molecules (*n* ∼ 14) when compared to LyeTxI (*n* ∼ 9) (Wieprecht et al., 2000). The higher entropic contribution observed for LyeTxI-b (19.8 cal mol^−1^ K^−1^) suggests greater desolvation of vesicle surface in comparison with LyeTxI (22.0 cal mol^−1^ K^−1^) during the peptide-membrane interaction. Although the peptides presented the same positive charge in the employed medium (pH 8.0), the enthalpic component for the peptide-membrane interaction of LyeTxI-b (−7,600 cal mol^−1^) was about 12 times greater than LyeTxI (−210 cal mol^−1^). These results are in accordance with the tridimensional structures of the peptides (Reis et al., 2018), which revealed a higher amphipathic helical conformation for LyeTxI-b when compared with LyeTxI, driving all positively charged residues to the hydrophilic face, with a more favorable arrangement for electrostatic interactions with a negative membrane surface.

**TABLE 2:** Thermodynamic parameters obtained after one binding experiment, non-linear adjustment for 25 μM LyeTxI or LyeTxI-b interaction with 20 mM POPC:POPG (3:1, mol:mol) LUV at 25°C.

Peptide	*n*	*k*_app_ (L mol^−1^)	Δ*G* ^o^ (cal mol^−1^)	Δ*H* ^o^(cal mol^−1^)	Δ*S* ^o^(cal mol^−1^.K^−1^)
LyeTxI	9	3.090 ± 150	-6.100	-210 ± 05	19.8
LyeTxI-b	14	7.270 ± 350	-14.200	-7.600 ± 50	22.0

Data were calculated according to Eq. 2 (see item *Isothermal Titration Calorimetry* section in this paper).

### Hydrodynamic Diameter and Zeta Potential Measurements

**FIGURE 3:**
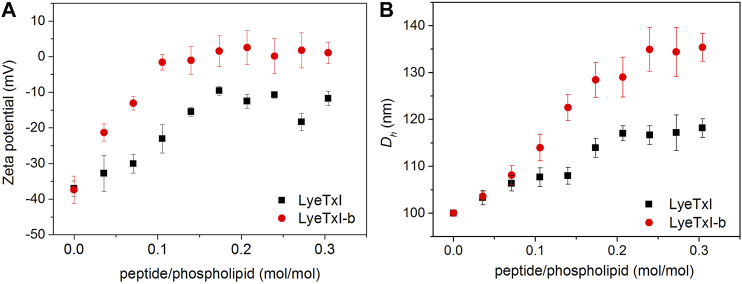
Graph of the variation of **(A)** zeta potential and **(B)** hydrodynamic diameter of 500 μM LUVs POPC:POPG (3:1, mol:mol) with the titration of 2.5 mM solution of LyeTxI (square in black) and LyeTxI-b (circle in red).

The interaction of the peptide with the bacterial membrane can disturb the phospholipid bilayer and, consequently, change its entire structural organization (Nguyen et al., 2011). Studies with several AMPs have revealed that the formation of supramolecular aggregates during the process of peptide-membrane interaction changes the volume and surface charge of the phospholipid vesicles (Abrunhosa et al., 2005; Silva et al., 2008). In this sense, we measured the hydrodynamic diameter (*D*_h_) and zeta potential (ζ) of POPC:POPG (3:1) LUVs (100 nm) before and after the addition of the peptides. Figure 3 shows the graphs of ζ and *D*_h_ variations, obtained from LUVs as a function of peptide/phospholipid molar ratio. Peptide addition led to an increase in both surface charge and *D*_h_ of the POPC:POPG vesicles due to the strong electrostatic interaction between the cationic peptides and the anionic phospholipid bilayer. Again, LyeTxI-b exerted stronger influence on the phospholipid vesicles properties when compared to LyeTxI. Molar ratios above 0.2 of LyeTxI-b led to a higher change in ζ (Δζ ≈ 40 mV) reaching the neutrality of the LUVs (Figure 3A). However, at comparable molar ratios LyeTxI caused a smaller change in the zeta potential (Δζ ≈ 30 mV) and the net charge of the LUVs remained negative. Likewise, whereas LyeTxI-b promoted an increase in the *D*_h_ of the LUVs of approximately 40 nm, LyeTxI increased the D_*h*_ in around 20 nm (Figure 3B). Again, these results point out the higher affinity of LyeTxI-b with anionic vesicles and, consequently, its greater effect on the disturbance of phospholipid bilayers.

### Inhibitory Activity on Planktonic Bacteria

Both peptides showed inhibitory activities against most of the resistant microorganisms tested at micromolar concentrations, except for *Serratia* spp. [SR7] and [SR10] strains (Table 3). In most of the assays, the peptides showed antibacterial activity at the same concentration range as the antibiotics, except for the samples of *A. baumannii* [34] and *E. coli* [7S], where such peptides were up to 200 times more active. For *A. baumannii* [34], *E. coli* [7S], *S. aureus* [20] and *P. aeruginosa* [33], LyeTxI-b showed two (for the 3 first strains) and four times (for last strain) higher activity when compared to LyeTxI. Surprisingly, *P. aeruginosa* strain [33] remained unaffected even at the highest concentration of LyeTxI tested. However, this same peptide was twice more active than its modified analog against *S. aureus* [8].

**TABLE 3:** Minimum inhibitory concentration (MIC) values for LyeTxI and LyeTxI-b in planktonic bacteria.

	LyeTxI MIC (µmol L^−1^)	LyeTxI-b MIC (µmol L^−1^)	Cefalexin MIC (µmol L^−1^)	Ciprofloxacin MIC (µmol L^−1^)	Chloramphenicol MIC (µmol L^−1^)	Gentamicin MIC (µmol L^-1^)
*Acinetobacter baumannii* [9]	2.80	2.88	>367.36	384	>395.52	>285.44
*Acinetobacter baumannii* [34]	2.80	1.44	>367.36	384	>395.52	>285.44
*Escherichia coli* [67]	2.80	2.88	22.96	192	>395.52	8.92
*Escherichia coli* [71]	11.20	11.52	11.48	12	>395.52	2.23
*Escherichia coli* [7S]	5.60	2.88	11.48	48	99.02	2.23
*Escherichia coli* [P10]	11.20	11.52	22.96	0.75	24.75	4.46
*Escherichia coli* [P11]	11.20	11.52	22.96	0.75	12.37	17.84
*Pseudomonas aeruginosa* [33]	>44.80	11.52	>367.36	6	49.51	17.84
*Serratia* sp*.* [SR7]	>44.80	>46.8	>367.36	1.50	>395.52	71.36
*Serratia* sp. [R10]	>44.80	>46.8	>367.36	1.50	>395.52	35.68
*Staphylococcus aureus* [8]	1.40	2.88	5.75	0.75	24.75	4.46
*Staphylococcus aureus* [20]	1.40	0.72	92	1.50	49.51	8.93

In previous works, we showed that the chemical changes in the structure of LyeTxI, giving rise to LyeTxI-b, confers a higher structural stability near the N-terminus and increases from 2 to 10 times the activity against planktonic bacteria (Reis et al., 2018). Nevertheless, in the present work, significant increases in the antimicrobial activity, rising from 2 to 4 times, between the native and the modified peptide, were observed only for *E. coli* [67] and [7S] and *S. aureus* [20].

The inhibitory activity of the peptides was also evaluated using fluorescein isothiocyanate (FITC). The peptides were labeled on the C-terminal leucine residue with FITC and their activity against *S. aureus* [20] was tested (Table 4). We observed a reduction in the inhibitory activities of FITC-labeled LyeTxI and LyeTxI-b of approximately 8 and 15 times, respectively. Our research group had already shown that the insertion of Hydrazinonicotinamide (HYNIC) at the N-terminal end of LyeTxI removed all its antimicrobial activity. However, labeling the C-terminal end did not change the peptide’s activity on *S. aureus* but decreased about 10x its activity on *E. coli* (Fuscaldi et al., 2016). In the present work, we expected that FITC tagging would not alter the activity of LyeTxI and its derivative LyeTxI-b, because through this tagging it would be possible to perform the internalization studies of these peptides by confocal microscopy, as previously described (Park et al., 2009; Joshi et al., 2010). As a perspective, we intend to study a marking position that favors these studies, without significantly altering the activity of the peptides.

**TABLE 4:** Minimum inhibitory concentration (MIC) values for LyeTxI and LyeTxI-b marked with FITC in planktonic *S. aureus* [20].

	MIC (µmol L^−1^)
LyeTxI	1.40
LyeTxI-b	0.72
LyeTxI (FITC)	23.04
LyeTxI-b (FITC)	11.2

### Death Time Curve

**FIGURE 4:**
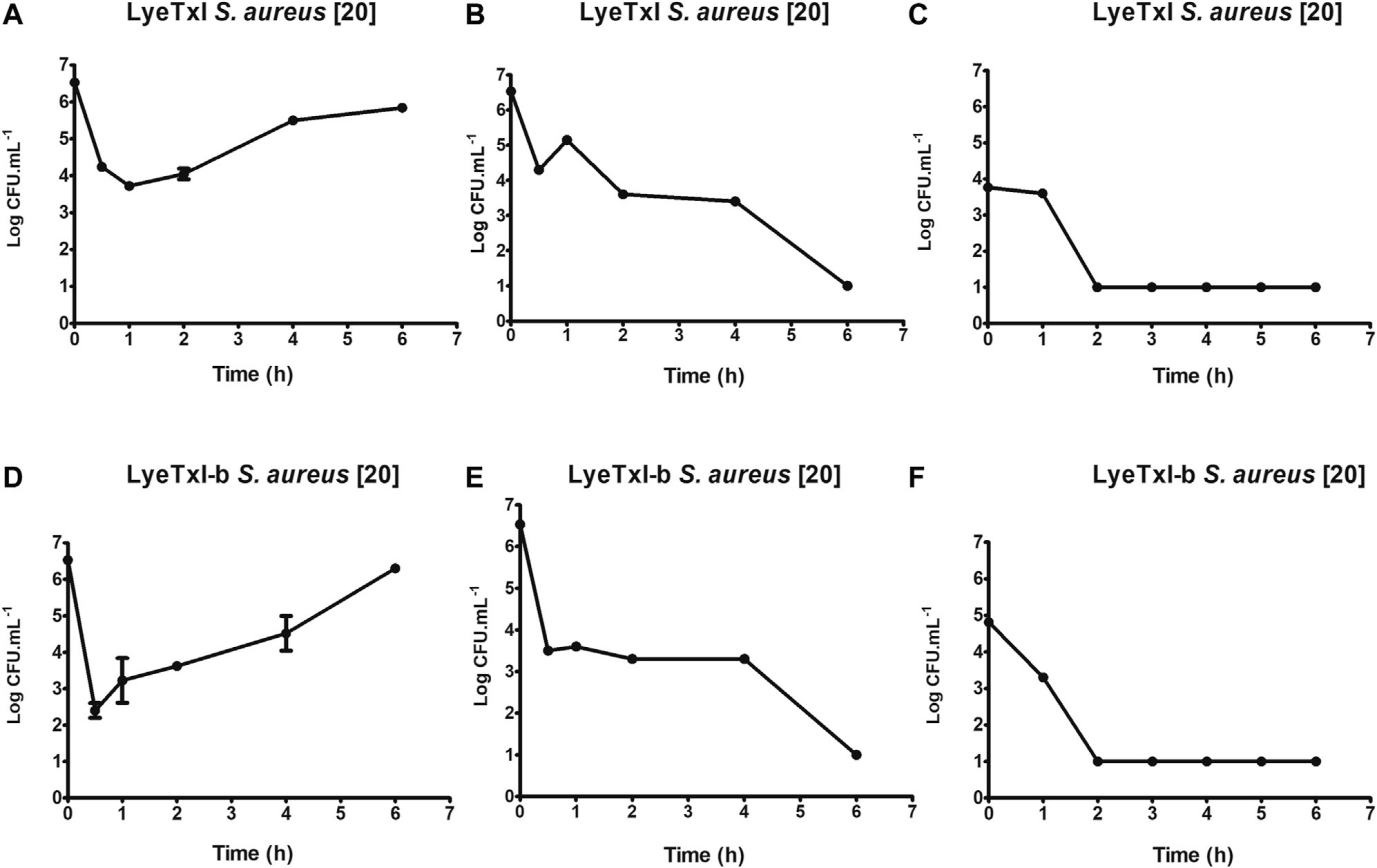
Kinetics of growth and time-dependent killing. *S. aureus* [20] was incubated with LyeTxI [½, 1 and 2 MICs = 1.4, 2.8, and 5.6 μmol L^−1^; Panels **(A–C)** respectively], or with LyeTxI-b [½, 1 and 2 MICs = 0.72, 1.44 and 2.88 μmol L^−1^; Panels **(D–F)**, respectively]. The incubations were at 37°C for 0.5 1, 2, 4, and 6 h. The density of viable cells was estimated at UFC.ml^−1^ using the microtip technique.

One of the initial steps for the characterization of a new drug is the determination of its time of action. As previously described, AMPs can have more than one mechanism of action, and the time of bactericidal activity is directly associated with the mechanism(s) of action. Our results for LyeTxI and LyeTxI-b reveal that they have similar patterns of dose-dependent activity, as shown inFigure 4. We started testing their activities at ½ MIC concentrations (LyeTxI in Figure 4A; LyeTxI-b in Figure 4D) and found a sharp drop in the number of viable bacteria within the first half hour. However, we saw that, after 1 h of exposure, the bacteria recovered and started their exponential growth. When we used the MIC concentration (LyeTxI in Figure 4B; LyeTxI-b in Figure 4E), we observed the same initial effect of accentuated death within the first 30 min, but they were followed by a “plateau” effect during 1–4 h of incubation with the peptides. The time of death in this experiment was defined between 4 and 6 h of exposure of the bacteria to the peptides. We also tested LyeTxI and LyeTxI-b at 2 x MIC concentrations (Figures 4C,F, respectively) and, for both peptides, the bactericidal activities occurred within 2 h of exposure. However, only LyeTxI-b treatment (Figure 4F) exhibited a sudden drop in bacterial levels within 1 h of exposure. Finally, the death curve shows that, in all experiments, LyeTxI-b had the same effect as the native peptide at a two times lower concentration.

The dose/effect relationship has already been described for other peptides and it has been associated with a cooperative effect between the peptide molecules to form pores in the membranes or even to have a detergent effect on them (Takeuchi et al., 2004). Joshi et al. (2010) observed that the cationic peptides under study showed bactericidal action in periods of 1–2 h of incubation and that the activity was associated with damage to the cell membrane. Ling et al. (2015) demonstrated that the peptide of their study, which acted by blocking lipid II - a precursor of peptidoglycan, has bactericidal action after 16 h of incubation. Our results present intermediate values between these previous works and demonstrate that, possibly, the peptides of LyeTxI family have multiple mechanisms of action, since their bactericidal action at the MIC concentration occurs during a period of time between 4 and 6 h of incubation.

### Oxidative Stress

**FIGURE 5:**
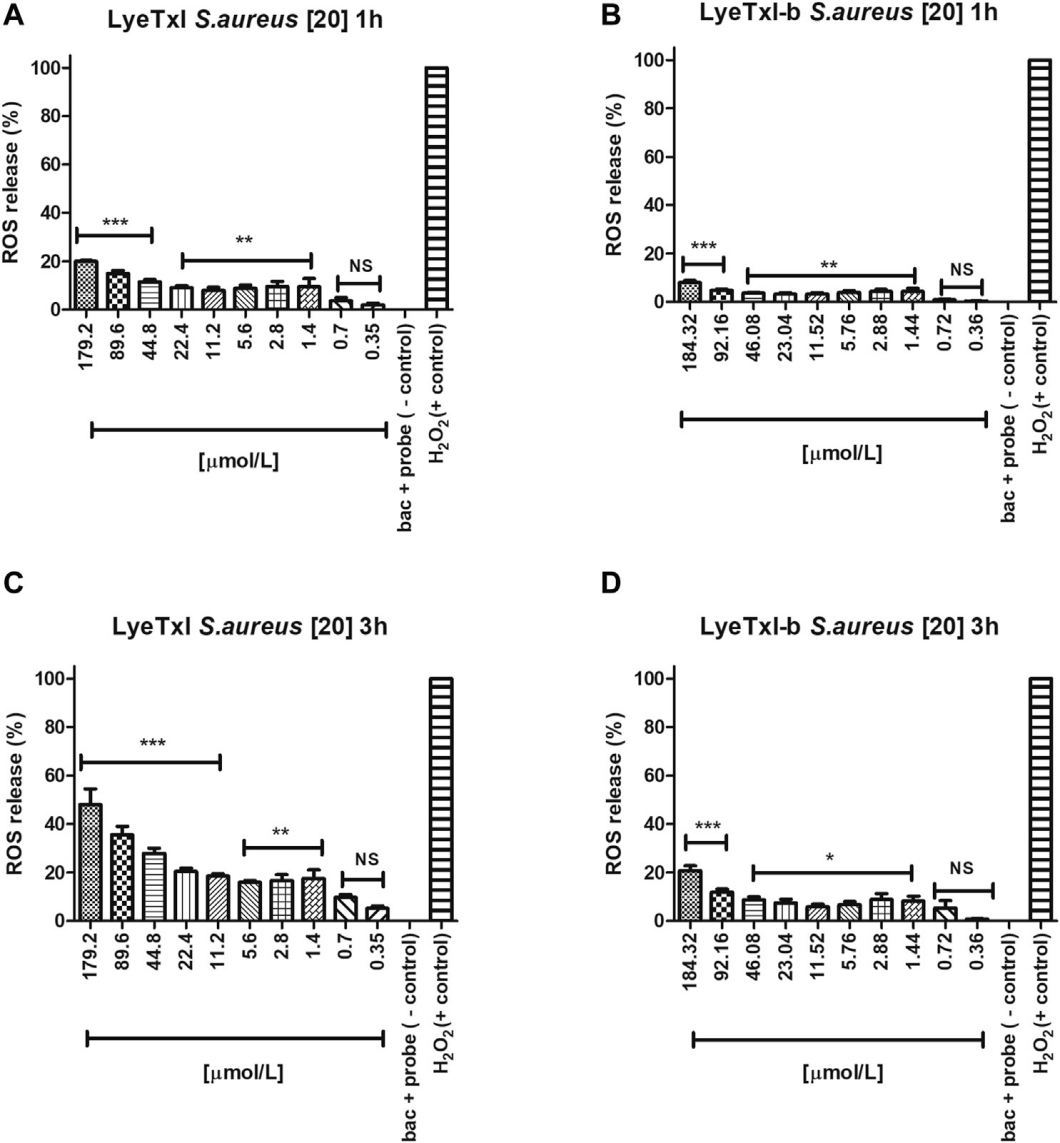
Quantification of oxidative stress. *S. aureus* [20] was incubated with the peptides, at 1 and 3 h, with different concentrations of LyeTxI **(A,C)** and LyeTxI-b **(B,D)**. Data were statistically analyzed by simple variance ANOVA followed by post-test Turkey, with *p < 0.05; **p < 0.01 and ***p < 0.001 in relation to the negative control, NS = not significant.

We investigated whether LyeTxI and LyeTxI-b would be able to induce the production of reactive oxygen species (ROS) after interacting with *S. aureus* bacteria at the same concentrations and exposure times (1–3 h) used in the MIC experiments, using the probe 2′, 7′-dichlorofluorescein-diacetate (DCFH-DA). This probe provides sensitive quantitation of ROS in response to oxidative burst since it emits fluorescence when oxidized. Thus, the fluorescence quantification can be used as an indirect measurement of intracellular oxidative stress (Wang and Joseph, 1999; Gomes et al., 2005). In Figures 5A,B, we observe that both peptides at the tested concentrations in a period of 1 h of incubation slightly increase ROS release in a dose-dependent way. However, at MIC concentrations, ROS release was lower or equal to 10% of the control (10 mM H_2_O_2 -_ considered as 100% release). After 3 h of incubation with the peptides, we observed the same dose-dependent effect on ROS release, but, surprisingly, LyeTxI (Figure 5C), at MIC concentrations, induced over 20% of ROS release, while at the highest tested dose ROS release was higher than 50%. On the other hand, the modified peptide, when incubated for 3 h, did not induce a release greater than 10% and, at the highest concentration tested, ROS release was near 20% (Figure 5D). Considering that an imbalance in the production and elimination of ROS causes oxidative stress, the cells must maintain oxidation-reduction ratio balance or the redox state, to deal with this effect (Lushchak, 2011). Therefore, our results show that the peptides tested act directly in this balance, in a dose-dependent relationship. However, the original and the modified peptides induced ROS release in a different proportion. LyeTxI showed a significant release depending on the dose, but the effects of LyeTxI-b were not significant.

### Antibacterial Activity in Biofilm

**FIGURE 6:**
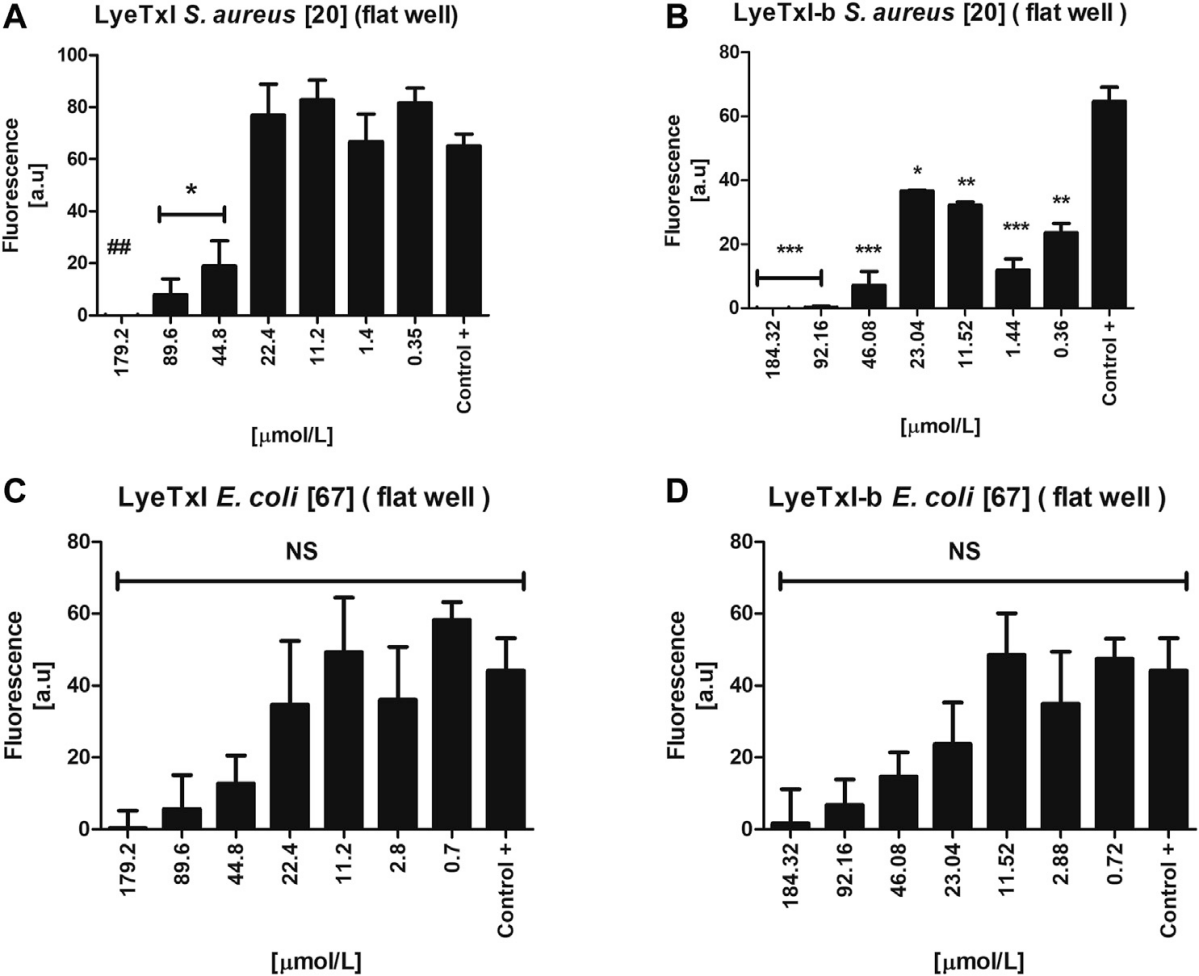
Activity in simple biofilm. Biofilm environment of isolated *S. aureus* [20] and *E. coli* [67] incubated with LyeTxI **(A,C)** and LyeTxI-b **(B,D)** at different concentrations of the peptides: 184.32 to 0.36 µmol L^−1^. Data were statistically analyzed by simple variance ANOVA followed by post-test Turkey, with *p < 0.05; **p < 0.01 ***p < 0.001 in relation to the control. NS = not significant.

We evaluated the activity of the peptides against the sessile cells of *S. aureus* and *E. coli*. LyeTxI was able to eradicate *S. aureus* biofilm (Figure 6A) only at the highest concentration tested (179.2 µmol L^−1^). On the other hand, LyeTxI-b was able to reduce *S. aureus* biofilms at the concentrations of 184.32, 92.16, and 46.08 µmol L^−1^. At the lowest concentrations tested, LyeTxI-b was still able to reduce the biofilm already formed, when compared to the control group (Figure 6B). For *E. coli*, although the peptides were not able to eliminate the biofilm, they promoted a reduction in cell viability at all concentrations tested, in a dose-dependent manner, although this was not statistically significant (Figures 6C,D).

Previously, our group demonstrated the potential ability of LyeTxI-b to reverse *in vivo* infections caused by *S. aureus* biofilm in a septic arthritis model, as well as to reverse keratitis caused by *S. aureus* in rabbit eyes (Reis et al., 2018; Silva et al., 2019). The literature has reported that some peptides have an antibiofilm activity at concentrations higher than MIC (Galdiero et al., 2019). Our present results show that both peptides present this pattern in *S. aureus* biofilm. It is also widely reported that the mechanism of action of AMPs in biofilm may be different from that seen in planktonic bacteria, because, in a biofilm environment, bacteria can increase the production and excretion of molecules, such as polysaccharides and DNA, in the biofilm matrix. These molecules, which are negatively charged, would interact with cationic AMPs, preventing their attachment to the bacterial membrane, which would require higher concentrations of AMPs for their activity, as we observed for *S. aureus* biofilm, or would reduce their activity, as shown in the results with *E. coli* biofilm (Chan et al., 2004; Mulcahy et al., 2008; Dostert et al., 2019).

### Evaluation of Possible Bacterial Resistance to Peptides

For the selection/induction of peptide-resistant mutants, we cultivated the bacteria in a medium containing sub-MIC concentration of the peptides. Thus, 10^5^ UFC ml^−1^ of methicillin-resistant *S. aureus* [20], at the beginning of the exponential phase, were incubated with 1/2 MIC of the peptides LyeTxI (0.7 μmol L^−1^) or LyeTxI-b (0.36 μmol L^−1^), at 24 h intervals for 20 passages. At the beginning and at the end of the experiment, MIC value was evaluated, to confirm whether resistant mutants had been selected. We performed two independent replicates of the experiment and we did not observe any changes in the initial or final MIC values for both peptides (Table 5). Although we observed a two-fold increase in MIC in the second replicate for LyeTxI-b, this difference was not considered significant.

**TABLE 5:** Minimum inhibitory concentration (MIC) values for LyeTxI and LyeTxI-b with sub-MIC for 20 days in planktonic *S. aureus* [20].

		MIC (μmol L^−1^)	
		Rep 1	Rep 2
LyeTxI	Start	1.40	1.40
	Final	1.40	1.40
LyeTxI-b	Start	0.72	0.72
	Final	0.72	1.44

As sub-MIC concentrations did not induce any peptide-resistant mutants of methicillin-resistant *S. aureus*, which is a promising result, we chose to carry out the experiment with progressively increasing concentrations of the peptides. The experiment started the same way as the previous one, but with a concentration of 1/16 MIC (LyeTxI 0.09 μmol L^−1^ and LyeTxI-b 0.046 μmol L^−1^) and at 24 h intervals the successive passage was performed with 100 x dilution from the previous one and the concentration of the peptides increased twice every passage. The experiments lasted 6 days and at the end we grew the bacteria on mannitol agar and performed the MIC test (Table 6). Again, there was no selection of resistant mutants for the peptides and, even reaching 2 x MIC concentration, the bacteria incubated with LyeTxI did not show any MIC alteration. We also observed that the bacteria incubated with LyeTxI-b had their MIC increased twice at the end of the experiment. However, this difference was not significant for resistance tests (MacLean et al., 2010; Andersson et al., 2016). Finally, we extended the experiments up to seven days of incubation, reaching the concentrations of 5.76 μmol L^−1^ for LyeTxI and 2.94 μmol L^−1^ for LyeTxI-b. We verified a full bactericidal action, confirmed with inoculation on mannitol agar medium.

**TABLE 6:** Minimum inhibitory concentration (MIC) values for LyeTxI and LyeTxI-b with increasing concentration in planktonic *S. aureus* [20].

		MIC (μmol L^−1^)	
		Rep 1	Rep 2
LyeTxI	Day 1	1.40	1.40
	Day 7	1.40	1.40
LyeTxI-b	Day 1	1.44	1.44
	Day 7	1.44	1.44

In the literature, there are few works on the resistance acquired by microorganisms to AMPs and for a long time the resistance to this promising class of drugs has been considered very difficult to occur (Shai, 2002). Most of the works that took the risk of doing experiments with acquired resistance used the first method used herein, where the bacteria are in contact with sub-MIC concentration in serial passages (Andersson et al., 2016). Although this method can select bacteria with mutations that favor their growth, we found that after twenty days of treatment it was not possible to select any mutants with our peptides. Ling et al. (2015) also demonstrated similar results with their Teixobactin peptide, where they could not select resistant mutants using sub-inhibitory concentrations for twenty days. Since it was impossible to isolate resistant bacteria in the pass-through method with the sub-MIC (1/2 MIC) concentration, we used a second incubation method with increasing concentrations of the peptides. Fehri et al. (2005) used these methods and demonstrated the possibility to select mutants. However, as discussed above, we did not obtain any selection of mutants, even when using this method. Therefore, the fact that the tested methicillin-resistant *S. aureus* is clinically relevant and that it was susceptible to the tested peptides with no induction of resistance in these experimental conditions confirms the great pharmaceutical/biotechnological potential of these peptides as new antimicrobial agents. Mishra and Bayer (2013) studying the features involved in methicillin-resistance of *S. aureus,* described that mprF locus in *S. aureus* is involved in the lysinylation of cellular membrane phosphatidylglycerol (PG) to generate the positively charged species lysyl-PG (LPG), and also promotes LPG translocation from the inner to outer CM leaflet. This mechanism may decrease the interaction of PAMs with the bacterial membrane. It seems that, in our experimental conditions it did not occur. This result also concurs to validate the use of POPG-containing vesicles in the present study, to evaluate the biophysical properties of the interaction of these peptides with membranes.

## Conclusion

In this work, we focused our studies on the correlation between LyeTxI-b structure and its antimicrobial activity compared with the original peptide LyeTxI. We found that both peptides were able to inhibit the growth of bacteria of medical importance, including those resistant to methicillin and carbapenems. Interestingly, we were not able to induce bacterial resistance to these peptides using the tested protocols. Moreover, we showed that the peptides induce internal production of ROS in a dose-dependent manner. We also found that the modified peptide LyeTxI-b has a slightly better effect than the native one in *S. aureus* biofilms, and they did not show significant activity in *E. coli* biofilms.

Our results show that small variations in the structure of AMPs (for example, the change in a single amino acid) can improve their activity, evidencing the great importance of exploring the engineering of these peptides for expanding their activities on various species and lineages of interest.

These studies may contribute to enforce the potential of AMPs for the development of new antibiotics, as an alternative for the treatment of infectious diseases caused by microorganisms, including those resistant to conventional drugs.

## Data Availability

The datasets presented in this study can be found in online repositories. The names of the repository/repositories and accession number(s) can be found in the article/Supplementary Material.

## Abbreviations

AMPs, antimicrobial peptides; BHI, brain heart infusion; CFU, colony-forming unit; CLSI, clinical and laboratory standards institute; DCFH-DA, dichlorofluorescein-diacetate; D_h_, hydrodynamic diameter; DLS, dynamic light scattering; FITC, fluorescein isothiocyanate; HYNIC, hydrazinonicotinamide; ITC, isothermal titration calorimetry; LMVs, large multilamellar vesicles; LUVs, large unilamellar vesicles; MH, Mueller-Hinton; MIC, minimum inhibitory concentration; PBS, phosphate buffered saline; POPC, 1-palmitoyl-2-oleoyl-sn-glycero-3-phosphocholine; POPG, 1-palmitoyl-2-oleoyl-phosphatidylglycerol; ζ, zeta potential; ROS, reactive oxygen species.
